# Attitudes towards genetic testing: The role of genetic literacy, motivated cognition, and socio-demographic characteristics

**DOI:** 10.1371/journal.pone.0293187

**Published:** 2023-11-15

**Authors:** Maxim Likhanov, Ilya Zakharov, Adeyemi Awofala, Olusegun Ogundele, Fatos Selita, Yulia Kovas, Robert Chapman

**Affiliations:** 1 State Key Laboratory of Cognitive Neuroscience and Learning, Beijing Normal University, Beijing, China; 2 Ural Federal University Named after the First President of Russia B. N. Yeltsin, Yekaterinburg, Russia; 3 Psychological Institute of Russian Academy of Education, Moscow, Russia; 4 Department of Biological Sciences, Tai Solarin University of Education, Ijebu-Ode, Nigeria; 5 Department of Psychology, Goldsmiths, University of London, London, United Kingdom; School of Medicine, University of California-Davis, UNITED STATES

## Abstract

Understanding reasons for why people choose to have or not to have a genetic test is essential given the ever-increasing use of genetic technologies in everyday life. The present study explored the multiple drivers of people’s attitudes towards genetic testing. Using the International Genetic Literacy and Attitudes Survey (iGLAS), we collected data on: (1) willingness to undergo testing; (2) genetic literacy; (3) motivated cognition; and (4) demographic and cultural characteristics. The 37 variables were explored in the largest to-date sample of 4311 participants from diverse demographic and cultural backgrounds. The results showed that 82% of participants were willing to undergo genetic testing for improved treatment; and over 73%—for research. The 35 predictor variables together explained only a small proportion of variance: 7%—in the willingness to test for Treatment; and 6%—for Research. The strongest predictors of willingness to undergo genetic testing were genetic knowledge and deterministic beliefs. Concerns about data misuse and about finding out unwanted health-related information were weakly negatively associated with willingness to undergo genetic testing. We also found some differences in factors linked to attitudes towards genetic testing across the countries included in this study. Our study demonstrates that decision-making regarding genetic testing is influenced by a large number of potentially interacting factors. Further research into these factors may help consumers to make decisions regarding genetic testing that are right for their specific circumstances.

## Introduction

Since the completion of the Human Genome Project in 2003 humanity has entered the Era of the Genome [1]. This new period is associated with the extensive use and development of genetic technologies, including genetic testing. For example, many specialists in the area predict that by 2030 in some countries the DNA of every newborn will be sequenced at birth [2]. This technology already exists and is becoming increasingly inexpensive: costing about $1000 for each full human genome sequence in 2020 [3], about $600 in 2023, and an anticipated $200 soon [4]. In addition, the time needed for genome sequencing has dramatically reduced from many days to around 7 hours [3, 5]. These trends mean that genetic testing, including direct-to-consumer, is becoming increasingly accessible.

Widespread availability of genetic testing may speed up the development of personalized medicine—optimized prophylactic or therapeutic solutions based on individuals’ genetic make-up [6], see e.g. a Precision Medicine Initiative [7]. Beyond medicine, genetic information can be applied in many contexts, including sports, education and the justice system [8, 9]. A growing body of research has begun to examine potential benefits and risks associated with diagnostic and predictive genetic testing [10–13].

### Willingness to undergo genetic testing

Relatedly, research has begun to examine people’s attitudes and views about genetic testing, indicating a trend from resistance to greater acceptance (see e.g. [14]). Several recent studies indicate that most people accept genetic testing for medical purposes. For example, in 2010 one study showed that 85% of 2000 respondents from a Russian urban population expressed positivity towards undergoing predictive genetic testing for preventable health conditions [15]. Similar results were found in another recent study, with almost 90% of participants from general populations in the UK, the USA and Russia expressing willingness to undergo genetic testing for improved treatment [16]. Another study with Romanian justice stakeholders, found that most judges and lawyers in the study expressed willingness to undergo genetic testing for medical purposes [17]. Yet another study showed that 71% of participants from a representative sample of 837 adult Qataris were willing to undergo genetic testing [18]. A high endorsement was also found in a large sample of 1500 Korean individuals from general public, 1500 cancer patients, 113 clinicians, and 413 researchers, with the majority of participants being positive towards genetic testing (from 88.5% among clinicians to 94.3% among patients [19]. A somewhat lower endorsement (63.8%) was shown by a study of Chinese individuals at high risk of breast cancer [20].

Research has also indicated that willingness to test for medical purposes is higher for treatable conditions and conditions with a clear family transmission patterns [17, 21, 22], and can be quite low in other cases [23]. Findings regarding attitudes towards genetic testing in non-medical contexts are much more mixed. For example, in one study, most legal professionals expressed willingness to provide DNA samples for research purposes [17]. In contrast, a study with university students in the USA found that only 11% were willing to donate DNA to research without reward, increasing to 50%—for payment [24]. People’s willingness to undergo genetic testing for other purposes is largely unexplored, including for family planning, career planning [e.g. taking up professional sports], and insurance decisions. Available literature suggests wide variability in such views [25–28].

### Genetic knowledge and willingness to undergo testing

Understanding factors that shape attitudes towards genetic testing is an important agenda for the Genomic Era. Several studies found that people’s views on genetic testing are related to their genetic literacy [18, 29, 30]. For example, one recent study, with more than 5400 participants from several countries, showed that willingness to undergo genetic testing was positively correlated with genetic knowledge (B = .18; [16]). The same study also found that the general population has relatively low genetic knowledge and held some common striking misconceptions. For example, only two thirds (68%) of participants were aware of the polygenic (many genes involved) nature of complex traits (autism spectrum disorder and schizophrenia). This finding is in line with several recent studies that showed quite low knowledge in different samples, including pharmacy students [21, 31]. Low knowledge of basic genetic concepts might lead to under-informed choices in relation to undergoing genetic testing, receiving consultation on genetic-related matters, or using off-the-shelf genetic services.

Genetic knowledge, however, does not explain much of the variance in people’s decision regarding genetic testing, as evidenced in the low correlation between them (e.g., [16]). This is because people are not passive recipients of scientific knowledge regarding genetics, but rather engage with it in a motivated fashion (see [32], for review). In other words, people’s judgments about genetic testing are based on rational considerations (’cognition’), as well as on their beliefs, attitudes and values (’motivated cognition’) [33]. It has been shown that motivated cognition factors can affect legal judgments in courts [33], political judgments [34, 35], and the use and interpretation of empirical research itself [36]. In the case of genetic testing, considerations can include risks related to access to health insurance and privacy, suspicions about hidden political/economic agenda behind genetic studies, concerns about misuses of genetic information, etc. (e.g. [16, 37]).

### Motivated cognition and willingness to undergo testing

Motivated cognitions relevant to genetic testing can also include beliefs regarding the malleability of different traits and an individual’s control over them. This is because such beliefs may influence one’s evaluations of usefulness of genetic information. For example, people’s beliefs regarding ‘free will’ have been linked to such phenomena as pursuit of self-directed goals, level of prosocial and aggressive behavior, autonomy and conformity, self-efficacy and perceived capacity [38]. In public health, deterministic beliefs were shown to impact perceptions of disease risk and inclination to engage in medical evaluations, prophylactics, and treatments (see [39] for review). For example, participants’ perception of a condition as being genetic was linked to: greater expectations on the effectiveness of genetic testing and related technologies [40]; reduced optimism for treatment and more willingness to seek medically intensive treatments [41, 42]; and decreased efforts to manage diseases such as diabetes with lifestyle changes [43]. It is possible that people who believe they have a conscious control of their behavior (’free will’), may be more willing to undergo genetic testing—so that they can act on this information by introducing changes to lifestyle and other prophylactics. For example, research has shown that people who knew that type 2 diabetes is preventable by means of life style changes had higher inclination to undergo genetic testing [44].

The attitudes towards genetic testing may also depend on whether people believe that genes are involved in traits (see [45] for a thorough discussion). Recent advances in behavioral genetics have shown at least moderate effects of genes on practically all human traits [46, 47]. However, many people still hold misconceptions, such as believing that genes are not important for human behaviour, that genes are important only for some traits, or that only genes are important for behaviour. For example, one study has shown that 25% of participants believed that their destiny is written in their genes [16].

### Demographic characteristics and willingness to undergo testing

Research has also shown that attitudes towards genetic testing are influenced by demographic characteristics. For example, some studies show that younger people have on average a more positive attitude towards genetic testing and demonstrated higher interest in it [15, 48, 49]. In addition, males demonstrated slightly more positive attitudes towards testing [15, 50]. Attitudes towards genetics can also be culturally informed [51]. Some cross-cultural differences in attitudes towards genetics have been found, including in views regarding general moral issues of self- and society-responsibility [52]; and specific questions of prenatal diagnostics and reproductive technologies [53]. For example, one recent study [54] has shown cross-country differences in endorsement of genetic testing before pregnancy by medical students: 96% of participants in Israel and 40% in Croatia disagreed with the idea that "Screening for reproductive risks in prospective parents is wrong". Another study found differences in awareness and attitudes concerning genetic testing across regions within one country (USA), suggesting some influences from local sociocultural environments [55]. Yet another study found that African Americans, compared with White Americans, anticipated fewer negative consequences of genetic testing identifying potential health-related problems [56]. A cross-cultural approach towards genetic testing and attitudes towards it allows to shed light on cultural/historical specifics that potentially might affects willingness to undergo testing. For example, consanguinity is common in south Asian and Arab societies (e.g. in Pakistan prevalence of consanguinity has been reported as high as 80% due to marriages within caste groups), which increases risk of genetic diseases (e.g. colour vision impairment [57] or β-thalassemia [58]). This may increase willingness to undergo genetic testing in these populations.

The interrelations between genetic knowledge, concerns about genetics, demographic characteristics, and willingness to undergo testing appear to be complex. For example, several studies reported gender differences in genetic knowledge, but results differed depending on the sample. In some studies, lower genetic knowledge levels were found in males [59, 60]; whereas other studies found lower levels in females [16, 61]. Moreover, some research suggested greater knowledge about biotechnology is associated with lower pessimism about biotechnology for men and with greater pessimism for women [61]. The age-related effects on genetics knowledge are also mixed. Numerous studies found that higher levels of genetic knowledge, especially among young adults with higher education levels, are associated with more favorable attitudes towards genetic testing, e.g. for chronic disease [62, 63]. However, this association was not found in some studies [48, 50]. For example, in one study, people aged 45–60 years and with less education showed most interest in genetic testing for heart disease [64].

Moreover, greater genetic knowledge may result in more concerns in some contexts. For example, in one study, women who disagreed with statements like “genetic information should be used to enable parents to choose physical and mental characteristics of their children” had higher genetic knowledge [65]. One qualitative study with 4 focus groups showed that after an open discussion of potential positive and negative implications of predictive genetic testing, a quarter of the participants initially interested in having a test changed their mind [66]. Another study [67] showed that well-informed participants had more critical attitudes towards morally or socially sensitive applications of genetics (e.g., genetic engineering). Yet another study showed that results of genetic testing and information provided after it might affect attitudes towards testing, with 91.6% of patients with negative results of BRCA 1/2 gene test and 100% of patients with positive results were willing to recommend family members to participate in the cancer screening program [20].

### The current study

Gaining further insights into sources of attitudes towards genetic testing will help people to make informed decisions regarding genetic testing in specific circumstances [32]. The present study explores the multiple drivers of people’s attitudes towards genetic testing, as well as interrelations among these factors. Using the International Genetic Literacy and Attitudes Survey (iGLAS) [68], we collected data on: 1. willingness to undergo testing (for improved treatment and for research); 2. genetic literacy—knowledge about the basic principles and current state of genetics; 3. motivated cognition—personal concerns about the benefits of testing and future applications of data, general trust in governmental research institutions or insurance companies; and 4. demographic and cultural characteristics—gender, age, level of education, country of residence, country of secondary education, occupation, and religiosity. These variables are explored in the largest to-date sample (N = 4311) of participants from diverse demographic and cultural backgrounds.

## Methods

### Participants

**Fig 1:**
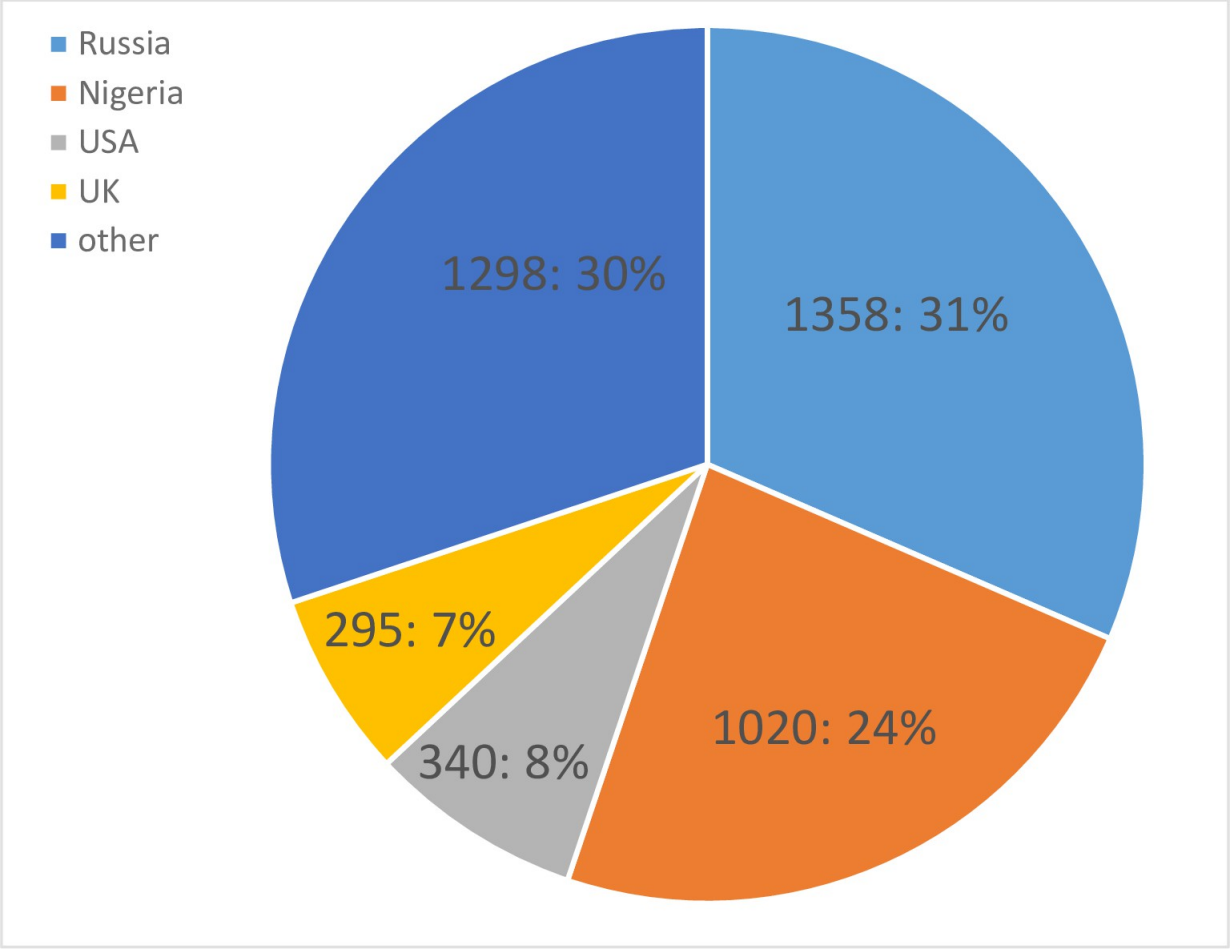
Pie chart for N of participants from different countries. Participants were reached through social media and Reddit AMA. Targeted collections were also carried out through higher education institutions in Nigeria, Russia, and the UK. Participants were recruited on a rolling basis over a period of 3 years, largely before Covid-19 pandemic. The targeted collections also happened before the pandemic.

The total sample included 5238 participants from 86 different countries. Participants had to be 18 or older, with no upper age limit. Eighty-six percent of the participants had either completed or were working towards an undergraduate degree or higher. The number of participants varied across different analyses, as not all participants answered all questions. After all data exclusions, including outliers deletion, and list-wise deletion (needed for factor analysis of Opinions), data from 4311 participants were analyzed. As most of participants came from Russia, Nigeria, USA and UK (more than 295 participants in each; See Fig 1), we run an additional analysis for these 4 countries, exploring differences in genetic testing attitudes across them. Other countries had smaller samples, with 6 countries having a sample size from 48 to 256 participants; 35 countries–from 2 to 39, and 15 countries–only 1 participant (see S1 Table for Ns of individual countries)

### Measures

We used the International Genetic Literacy and Attitudes Survey (iGLAS). The current version of iGLAS is available in 9 languages (Albanian, English, French, Italian, Japanese, Persian, Romanian, Russian, and Spanish). A short sample of the English language version of iGLAS can be found at https://goldpsych.eu.qualtrics.com/jfe/form/SV_9zOfCcGhht7qwy9. Information on the validation of iGLAS can be found in [68]. The latest version of iGLAS can be found at http://tagc.world/iglas/.

In the present study we used 2 items to explore willingness to undergo genetic testing and 37 items—as potential predictors of the willingness to undergo genetic testing. The predictors represented three groups of potential sources of individual differences: 1) genetic literacy; 2) motivated cognition; and 3) demographic characteristics.

#### Outcome

*Willingness to undergo genetic testing*. Two questions explored participants’ willingness to undergo genetic testing, using 7-point Likert scales (Response options: 1- “Very unlikely”, 2 - “Unlikely”, 3 - “Somewhat unlikely”, 4 - “Neutral”, 5 - “Somewhat likely”, 6 - “Likely”, and 7 –“Very likely”):

1. “*Would you take a genetic test if it allowed you to have improved treatment (for example*, *medication with fewer side effects)*” (Henceforth **Test for treatment**);
2. “*Would you be willing to give a sample of your DNA for scientific research if your data are stored anonymously*?” (Henceforth **Test for science**).

#### Predictors

1. **Genetic literacy** was measured with 20 items.
  The questions were developed to assess a basic functional level of genetic knowledge. An example item with 4 response options being: “*Which of the following 4 letter groups represent the base units of DNA*: *1) GHPO; 2) HTPR; 3) GCTA; and 4) LFWE*?”. More details about the genetic knowledge items can be found in [16]. In the present study the total **Genetic Knowledge** score was obtained by summing up correct responses for each item.
2. **Motivated cognition** was assessed with 10 items.
  8 of the items tapped into participants’ concerns about genetic testing. Participants were asked to answer whether they have any of the 8 concerns in response to the following question: “*In deciding whether to take a genetic test*, *which of the considerations below apply to you”* (See Table 1). Multiple responses could be chosen. There was an additional– 9th, free text option in this question: “Other”. More than 95% of the participants did not state any “other” consideration, thus, this question was not included into further analysis.

**Table 1:** Response options to the iGLAS “concerns” question.

	Concern	Percentage of participants, having a particular concern	Factor loadings	Factor
Q1	I don’t know who will have access to that information	38%	0.6	**Data security**
Q2	I don’t know whether the data will be stored securely	32%	0.7	**Data security**
Q3	I would rather not know of any potential debilitating diseases in my future	90%	0.4	**Health issues**
Q4	I’m not interested	11%	NA	NA
Q5	I’m worried that I might find out something about myself I would rather not know	18%	0.5	**Health issues**
Q6	I would not want to be labeled as having any deficiency	14%	0.3	**Health issues**
Q7	I’m worried some information about my physical or mental health could be used against me (e.g. employment; legal matters; obtaining insurance)	35%	0.5	**Data security**
Q8	I am concerned my data will be used for other purposes without my knowledge	41%	0.6	**Data security**

Note: Response options Yes/No were coded as 1/0.

Prior to the main analysis, a factor analysis was conducted on 8 concern items. Scree plot and the eigenvalues have shown that there are two concerns factors. Exploratory factor analysis with 2 factors and Oblimin rotation have shown that Q1, Q2, Q7 and Q8 loaded on a single Data security factor; and Q3, Q5 and Q6 loaded onto Health issues factor (see Table 1). Q4 did not load on any of the factors and was also excluded. **Data security** and **Health issues** variables were used as predictors in further analysis.

1 item tapped into views on genetic influences on behavior: “Destiny is written in our genes” (**Destiny is written**). 1 item tapped into views on potential data usage violations **Mistrust in research**: “I do not trust research institutions in my country because they might misuse data obtained from participants”. A 7-level Likert scale (from “Strongly disagree” to “Strongly agree”) was used for both questions.

**3. Demographics** included the following 5 characteristics: **Gender, Age, Country of residence, Level of education** (on a self-report 7-point scale from “no school certification” through to “post-doctoral”), and level of **Religiosity** (on a self-report 10-point scale, varying from “Not at all” to “Very religious”).

### Procedure

The study was approved by the Goldsmiths Department of Psychology Ethics Committee (ref: PS101016RCS). All the analyses presented in this paper were based on data accessed after ethical approval for the study was provided by the ethics committee of the psychology department at Goldsmiths, University of London on 10/10/2016. Participants answered iGLAS items online, in their own time and place; or at their University. The data were fully anonymous. Informed consent was implemented at the beginning of the survey. Participants were asked to accept the following statements: My participation in this study is voluntary; I am over 18; I may withdraw from this research at any time and for any reason; I may omit any questions I do not wish to answer; All data will be treated with full confidentiality and, if published, it will not be identifiable as mine. For those who did not endorse any of the statements, the survey discontinued.

#### Statistical approach

The data were analyzed with R language for statistical programming and R studio platform (R Core Team, 2017). The regression and correlation (Pearson) analyses were performed with in-built R functions “lm” and “cor”, the factor analysis was performed with the ‘psych’ package (https://personality-project.org/r). Significance level was set to p < 0.05. Mixed-effect modelling was performed with lme4 package [69]. Compliance with normality and linearity assumptions was ensured for all analysis. Individual answers that exceeded a ± 3 SD level were considered outliers and deleted. Significance level was set at p < .05 threshold. The R code used for all analysis is available at: https://osf.io/nex76/

## Results

### 4.1 Descriptive statistics for the full sample

The descriptive statistics for the study variables are presented in Table 2. 2545 females (59.03%) and 1606 males participated in the study. 30 participants identified as being non-binary, 29 participants decided not to disclose their gender and 101 participants did not provide information on their gender. Only data from male and female groups were used in regression analyses, as the other groups were too small. Most of the sample were working towards or had at least an undergraduate degree (70.5%) at the time of completing iGLAS, with extra 8.7% working towards or holding an MSc and 6.9%—a PhD degree. Age for some participants exceeded +3 SD threshold (~ 62.09), however we decided to include them in further analysis too. Frequencies for **Country of residence** can be found in SOM Table 1.

**Table 2:** Descriptive statistics for the 8 study variables.

Variable name	n	mean	sd	median	min	max	skew	kurtosis
**Test for treatment**	4311	5.62	1.63	6	1	7	-1.36	1.14
**Test for science**	4298	5.22	1.81	6	1	7	-0.99	-0.06
Genetic Knowledge	4311	11.55	3.42	11	2	20	0.15	-0.68
Data Security*	4024	-0.01	0.86	-0.26	-1.17	1.73	0.67	-0.81
Health issues*	4024	0	0.66	-0.17	-0.83	2.52	1.51	2.15
Religiosity	3408	4.28	3.19	4	0	10	0.18	-1.16
Destiny is written	4308	3.02	1.66	3	1	7	0.46	-0.93
Mistrust in research	4304	3.78	1.78	4	1	7	0.06	-1.11
Age**	4158	26.75	11.78	22	18	80	2.04	3.92

Note: *Standardized values for factors extracted from EFA of “concerns” questions are presented

### 4.2 Willingness to undergo genetic testing

As can be seen from Table 2, the mean response for both willingness items was above 5 (out of 7), indicating considerable willingness to undergo genetic testing. A paired sample t-test showed that willingness to undergo **Test for treatment** was slightly higher than **Test for science** (Cohen’s d equals 0.21).

We also computed percentages for each response option, showing that 81.64% of the respondents chose “Very likely”, “Likely”, and “Somewhat likely” for **Test for treatment** and 72.78%—for **Test for science** questions.

### 4.3 Associations among the study variables

Zero-order correlations (Pearson) among all study variables are presented in Table 3. The two outcome variables were moderately correlated (r = .42), suggesting that participants who were willing to undergo genetic testing for improved medical treatment were also on average more likely to undergo genetic testing for research purposes.

**Table 3:** The relationship between study variables.

Variable	Test for treatment		Test for science		Genetic Knowledge		Data Security		Health issues		Religiosity		Destiny is written		Mistrust in research		Age		Gender
**1. Test for treatment**	—																		
**2. Test for science**	.42	***	—																
3. Genetic Knowledge	.20	***	.21	***	—														
4. Data Security	.09	***	.02		.24	***	—												
5. Health issues	.03	*	.05	**	.09	***	.29	***	—										
6. Religiosity	-.13	***	-.09	***	-.18	***	-.17	***	-.06	**	—								
7. Destiny is written	.14	***	.10	***	.05	**	.06	***	.07	***	-.04	**	—						
8. Mistrust in research	-.05	**	-.11	***	-.11	***	.18	***	.07	***	.08	***	.04	**	—				
9. Age	.09	***	.12	***	.35	***	.17	***	.01		-.11	***	.10	***	-.11	***	—		
10. Gender#	-.09	***	-.10	***	-.21	***	-.07	***	.05	**	.11	***	-.02		.04	**	-.20	***	—
11. Education level	.02		.06	***	.22	***	.04	*	.02		.08	***	-.00		-.06	***	.25	***	-.02

Note: * p < .05

** p < .01

*** p < .001; #Gender was coded as follows: 1—Male, 2- Female, 3- non-binary explaining e.g. a negative correlation with genetic testing–males had higher willingness to undergo testing compared to females.

Willingness to undergo genetic testing in both contexts was positively correlated with **Genetic knowledge** (r = ~.20), as well as with **Age** (r = ~.10), **Education level** (r = ~.06) and deterministic views (**Destiny is written**; r = ~.10); and negatively correlated with **Religiosity** (r = ~.11) and **Mistrust in research** (r = ~.08). **Data security** and **Health issues** concerns showed only negligible correlations with willingness to undergo testing. **Educational level** did not correlate with willingness to undergo **Test for treatment** and only negligibly with **Test for science**.

### 4.4 Statistical predictors of willingness to undergo genetic testing in the full sample

We tested two step-wise regression models, exploring predictors of willingness to undergo **Test for treatment** and **Test for science**. **Genetic knowledge**, **Destiny is written, Religiosity, Mistrust in Research** and **Gender** were positively associated with **Test for treatment**, together explaining approximately 7% of the variance. The same set of significant predictors were shown for willingness to undergo **Test for science**, together explaining 6.3% of the variance (Table 4).

**Table 4:** Results of stepwise regression for to willingness to undergo genetic testing questions.

	Test for treatment		Test for science	
Variable	β	95% CI	β	95% CI
(Intercept)	.00	[-.03, .03]	.00	[-.03, .03]
Genetic Knowledge	.17**	[.13, .20]	.18***	[.13, .20]
Destiny is written	.12**	[.09, .16]	.08***	[.08, .16]
Religiosity	-.09**	[-.13, -.05]	-.04**	[-.13, -.03]
Mistrust in research	-.04*	[-.08, -.01]	-.08***	[-.09, -.01]
Gender	-.04*	[-.07, -.00]	-.04*	[-.07, -.00]
Model’s R2	.069**		.063**	

Note. * indicates p < .05

** indicates p < .01

*** indicates p < .001; Stepwise regression excluded **Age**, **Education level**, both concern factors (**Data security and Health issues**) and **Country of residence** for both outcomes (p-level for removal = 0.1).

To control for effect of **Country of residence**, we ran a similar model (predicting **both outcomes** from all study predictors) but with a random intercept for **Country of residence**–random slope model did not fit because of small sample sizes for some countries (15 countries with N = 1). The mixed model analysis showed a result similar to stepwise regression (i.e. same predictors being significant), explaining 6.2% in **Test for treatment** with extra 0.3% when random effects were accounted for. For **Test for science** the mixed model analysis showed an almost similar result, with fixed effects of **Genetic Knowledge, Destiny is written, Religiosity, Mistrust in research** and **Gender** being significant. The only extra predictor was **Data security** (with very small effect– 0.02). This set of predictors explained 7.1% in **Test for treatment** with no extra addition from random effects. Extra details on the analysis are available from authors on request.

### 4.5 Country differences in willingness to undergo genetic testing

We next examined potential cross-cultural differences, re-running the analyses for four countries with the biggest samples separately: participants from Russia (N = 1358), Nigeria (N = 1020), the USA (N = 340) and the UK (N = 295). Table 5 presents descriptive statistics and ANOVA results for all variables divided by country. Post-hoc comparisons for all variables are available in S2 Table in SOM.

**Table 5:** The descriptive statistics and the effect of the country factor on the study variables.

	Russia		Nigeria		USA		UK		One-way ANOVA results
	Mean	SD	Mean	SD	Mean	SD	Mean	SD	Partial eta^2
**Test for treatment**	5.42	1.69	5.25	1.82	5.88	1.45	5.92	1.26	.02***
**Test for science**	4.86	1.95	5.00	1.88	5.61	1.59	5.78	1.33	.03***
Genetic Knowledge+	50	12	55	15	78	14	73	17	.33***
Data security	-.05	.78	-.37	.56	.66	.93	.2	1.01	.14***
Health issues	-.06	.59	-.07	.63	.07	.68	.06	.76	.01***
Religiosity	3.42	2.66	7.08	2.15	3.48	3.14	2.73	2.97	.32***
Destiny is written	3.11	1.51	2.58	1.86	3.32	1.59	3.23	1.5	.03***
Mistrust in research	3.87	1.49	4.32	2.01	4.02	1.8	3.15	1.62	.03***
Age	20.11	4.46	22.47	4.9	38.55	15.02	43.73	15.99	.52***
Gender	1.77	.54	1.73	.46	1.49	.52	1.53	.5	.04***
Education level	3.80	.64	4.29	.69	4.32	1.18	4.37	1.11	.09***

Note. b represents unstandardized regression weights. +Genetic knowledge is in percentages correct.

*** indicates p < .001.

Our data showed that frequencies for willingness to undergo testing were quite different across the 4 countries, with small effect. For **Test for treatment**, the highest endorsement of testing (participants opting for “Very likely”, “Likely”, and “Somewhat likely”) was shown for UK (87.51%) and USA (86.46%), followed by Russia (78.69%) and Nigeria (74.11%). The pattern was the same for the overall endorsement of **Test for science**, with the highest endorsement in the UK (82.42%) and the USA (81.18%), followed by Russia (67.36%) and Nigeria (67.48%).

Of all predictors, three showed substantial average differences across the samples: for **Age**, UK and USA participants were considerably older than those from Nigeria and Russia; the overall **Genetic knowledge** was higher in UK and USA samples, compared to those from Nigeria and Russia; and **Religiosity** was higher in Nigeria than in the other 3 countries. There were some differences across the 4 countries in the Educational level, with Educational level for Russia being lower when in Nigeria, USA and UK; and no differences across the three (See S2 Table).

### 4.6 Statistical predictors of willingness to undergo genetic testing by country

The results of the separate step-wise regression analysis for each group are presented in Tables 6 and 7.

**Table 6:** Stepwise regression model, predicting Test for treatment for 4 countries.

	Russia		Nigeria		USA		UK	
	β	95%CI	β	95%CI	β	95%CI	β	95%CI
(Intercept)	.00	[-.06,.06]	.00	[-.07,.07]	.00	[-.12,.12]	.00	[-.14,.14]
Genetic Knowledge	.19**	[.13,.24]	.14**	[.07,.21]	.17**	[.04,.29]	ns	ns
Data security	ns	ns	.11**	[.04,.18]	-.16*	[-.30,-.02]	ns	ns
Health issues	.07*	[.02,.13]	ns	ns	ns	ns	-.24**	[-.39,-.10]
Religiosity	-.07*	[-.13,-.02]	ns	ns	ns	ns	ns	ns
Destiny is written	.12**	[.06, .18]	.13**	[.06,.20]	.18**	[.06,.30]	ns	ns
Mistrust in research	ns	ns	.10**	[.03,.17]	ns	ns	-.33**	[-.49,-.18]
Model’s R2	.063**		.064**		.125**		.169**	

Note. β represents standardized regression coefficients.

* indicates p < .05.

** indicates p < .01. ns–Non-significant predictor that were removed by step-wise procedure (p-level for removal = 0.1).

**Table 7:** Stepwise regression model, predicting Test for science for 4 countries.

	Russia		Nigeria		USA		UK	
	β	95%CI	β	95%CI	β	95%CI	β	95%CI
(Intercept)	-.00	[-.06, .06]	.00	[-.07, .07]	.00	[-.12, .12]	-.00	[-.09, .09]
Genetic Knowledge	.11**	[.05, .17]	.09*	[.01, .16]	.13*	[.00, .25]	.17*	[.03, .31]
Data security	ns	ns	ns	ns	-0.17*	[-0.30, -0.04]	ns	ns
Health issues	.12**	[.06, .18]	ns	ns	ns	ns	-.14*	[-.28, .00]
Religiosity	-.11**	[-.17, -.05]	ns	ns	ns	ns	ns	ns
Destiny is written	.08*	[.02, .14]	.09*	[.02, .16]	.16**	[.05, .28]	ns	ns
Mistrust in research	-.10**	[-.15, -.04]	ns	ns	-0.16*	[-.30, -.02]	-.25**	[-.39, -.12]
Model’s R2	.051**		.015**		.128**		.143**	

Note. β represents standardized regression coefficients.

* indicates p < .05.

** indicates p < .01. ns–non-significant predictor that were removed by step-wise procedure (p-level for removal = 0.1).

None of the predictors of **Test for treatment** replicated across all 4 samples. Two predictors were significant in 3 samples (excluding UK): level of **Genetic knowledge** and the deterministic views (**Destiny is written**). For other predictors, there were differences across the countries. For example, greater **Mistrust in research** institutions and **Health issues** (greater concern regarding medical information) were moderately associated with less willingness to undergo genetic testing for treatment (**Test for treatment**) only in the UK sample. More concerns regarding **Data security** were associated with less willingness to undertake **Test for treatment** in the USA sample, and, surprisingly, with more willingness in the Nigerian sample. Greater religiosity was associated with less willingness to undergo **Test for treatment** only in the Russian sample. Overall, more variance was explained by the predictors in the UK and the USA (17 and 13%), than in Nigeria and Russia (~6%).

For the **Test for science**, the only significant predictor for all countries was **Genetic Knowledge**. Deterministic views (**Destiny is written**) was positively linked to **Test for science** in three countries (except the UK). **Mistrust in research** was negatively linked to **Test for science** in the three countries (except Nigeria). The pattern of results was somewhat different for other predictors. For example, **Data security** was negatively associated with **Test for science** only in the USA sample. **Health concerns** were negatively associated with willingness to **Test for science** in the UK; and positively–in Russia. **Religiosity** was negatively associated with **Test for science** only in the Russian sample. Overall, more variance was explained by the predictors in the USA and the UK (13 and 14%), than in Nigeria and Russia (2 and 5%).

## Discussion

The current study explored attitudes towards genetic testing and the sources of individual differences in such attitudes in the largest to-date sample of participants from diverse demographic and cultural backgrounds.

Our data showed that 82% of the 4311 participants were willing to undergo genetic testing for improved treatment; and 73%—for research. This finding is in line with previously shown positive attitudes towards genetic testing in samples from different countries [15, 16, 51, 70–72]. Consistent with previous findings, participants were more willing to test for treatment than for science. One explanation for higher endorsement of testing for improved treatment might be that people perceive testing for improved treatment as beneficial for them and their relatives (“benevolent”); compared with “abstract greater good” (“altruistic”) testing for science. A similar pattern of results was shown in a study that investigated motivation to donate blood [73]. Blood donors were more willing to donate blood when exposed to a benevolent message (selfish + societal benefits) rather than an altruistic one (societal benefits only). The differences may also reflect a preference for immediate benefit (improved treatment) over a “delayed gratification” [74]–willingness to contribute to a later reward (potential better diagnostics and treatments in the future). However, differences in percentages between the two questions are quite small, suggesting that people view both testing options as potentially useful. Interestingly, the correlation between the two purposes of testing was only moderate [42], suggesting that many people endorse only one of the contexts and not another, or endorse them to a different extent. As a whole, these results highlight complexities of the processes underlying public understanding of genetic science and reasons for why people opt for genetic testing, which may be of interest to stakeholders and policymakers.

Analysis of willingness to undergo testing in separate countries with the largest samples in the current study (Russia, Nigeria, the USA and the UK) showed lower willingness in Russia and Nigeria. Several sample characteristics could have contributed to the observed differences. For example, USA and UK samples were on average older (Partial eta squared equals .52) and had higher genetic literacy (.33). In addition, USA and UK have higher GDP [75], which may also be relevant in terms of genetic testing advancements and accessibility. Differences in educational systems, mass-media coverage of genetic discoveries and overall scientific literacy levels across countries may all also contribute to the differences in genetic testing attitudes and warrant further investigation.

This study also explored factors that might explain individual differences in people’s willingness to undergo genetic testing in the overall sample and within the four countries. We examined genetic knowledge, motivated cognition (beliefs and concerns), and demographic characteristics—as potential predictors. Overall, the investigated factors explained little variance in willingness to undergo genetic testing: around 7% in testing for treatment and 6% for research. The effects of individual predictors were weak, with genetic knowledge being the strongest positive predictor for both treatment and research. Contribution of genetic knowledge to willingness to undergo testing is expected and in line with previous research [16, 18]. Our results support suggestions that a lack of knowledge is one of the most important barriers to the acceptance of genetic technologies [76], potentially because it prevents comprehension of potential testing results [77].

On the other hand, genetic knowledge explained only a little variance in willingness to undergo genetic testing, which indicates the complex nature of this association. Some research suggested that greater genetic literacy is associated with greater reported fear and uncertainty about the implications of genetic testing; and, in contrast, lack of understanding and unreasonable expectations may lead to unwarranted enthusiasm about testing and unrealistic hopes about its results [65, 78]. Our data showed that, on average, participants correctly answered 60.4% of the genetic knowledge questions, which indicates insufficient knowledge given that the questionnaire tapped into basic knowledge regarding genetics. More nuanced research is needed to understand whether greater genetic knowledge may allow people to assess pros and cons of testing in every situation and undergo testing when it can actually be beneficial to them.

In line with our prediction, the belief that ’one’s destiny is written in one’s genes’ was positively associated with willingness to undergo genetic testing (.08-.12). Given that genetic testing can provide probabilistic prediction of future life outcomes (e.g. educational achievement; [79]), it seems natural for people who believe that genes are important for different traits and behaviours to want to know what is ’written there’ and potentially to alter ‘destiny’. People can introduce various changes to their lifestyles to ameliorate the potential negative effects of genes on their behaviour and health. For example, they can choose to get some extra reading classes and other educational interventions for a child, if a predisposition to reading problems is identified via genetic testing [80]. In the future, gene editing advances may also enable prevention of diseases or improved treatments, with the first cases of genetic therapies already at the clinical trials stage [81–83].

Further analysis of predictors for willingness to undergo genetic testing in UK, USA, Nigeria and Russia separately showed both similarities and differences across the four countries. Genetic knowledge and belief in ‘genetic destiny’ predicted willingness to test for treatment in 3 of the 4 countries. The absence of these effects in the UK can be explained by specific characteristics of the UK sample in this study. For example, this sample was the oldest out of the four (Mage = 43.73) and had the highest average education level (4.37). However, the overall effect of Level of education was small (eta squared equal to .09). Moreover, the UK participants showed the highest overall willingness to undergo test for treatment and the narrowest distribution of scores around the mean. This restricted variance may have led to the observed lack of prediction.

Several predictors that were not present in the overall sample emerged in the analysis of data for separate countries. For Test for treatment, data security was a significant predictor in Nigeria and the USA; while health-related concerns—in Russia and the UK. One of the strongest predictors in the UK sample was mistrust of research institutions (-.33), which is in line with some previous studies [44]. Such mistrust may stem both from people’s personal experiences, as well as awareness of infamous violations of research ethics in previous studies. For example, studies that might have negatively affected attitudes towards research institutions may include the “Tuskegee Syphilis Study”, “Stanford Prison experiment”, and many other across the world (e.g. [84]). Beyond mistrust of research institutions, people’s mistrust of various kinds of official institutions (police, courts, the government) may influence the decisions to undergo testing [85]. Quite a similar pattern emerged for the willingness to test for science, with genetic knowledge being a significant predictor for all countries; and belief in ‘genetic destiny’–for three out of four countries (except UK). Mistrust of research institutions was also a significant predictor in three out of four countries (except Nigeria).

Data security concern was a significant negative predictor of willingness to test for science only in the USA. This negative effect on a decision to undergo genetic testing is expected, as recently there have been many public outcries regarding data protection and storage, including public cases of companies selling genomic data for profit. For example, 23andMe sold the data of participants to GlaxoSmithKline for $USD300 million [86]. Given that genomic data can reveal a person’s life history, ancestry and genetic predispositions, using such data poses serious ethical, social and legal concerns, including in the sectors of employment, health insurance and justice [87, 88]. A potential genomic data breach might cause serious harm, including discrimination in health insurance [89]. It is not clear why Data security concerns were not a significant predictor in the other samples, as data security risks are well known. For example, one recent study that analyzed the NHS Digital audits found that in the past year 33 UK organisations, including GlaxoSmithKline and Imperial College London, were audited and every one had breached data sharing agreements, with hundreds more inspected and found in breach since audits began in 2015 [90]. In our study, fewer data security concerns were reported by participants from Russia, Nigeria and UK, compared with the USA (who also showed the highest genetic knowledge). It might be that data security concerns are not linked to willingness to undergo testing in Russia, Nigeria and UK as participants are underinformed regarding consequences, including negative ones, of genetic testing. This explanation is in line with one previous study that showed well-informed participants had more critical attitudes towards applications of genetics [67]; and is indirectly supported by a relatively high correlation between genetic knowledge and data security concerns (.24). Further research is needed to investigate complex interactions across knowledge, concerns, and attitudes towards testing.

Contrary to the pattern obtained for the whole sample, health-related concerns were a significant negative predictor in the UK; and a significant positive predictor in Russia for testing for treatment and science. A negative effect of health-related concerns is in line with previous studies that showed people worrying about gene-based health information. For example, US citizens were found to be afraid that genetic test results could be used to discriminate against those with a genetic predisposition for illness [37]; or that genetic testing could result in people being labeled as having “good” or “bad” genes [76]. Previous studies also found negative associations between attitudes towards genetic testing and fear that genetic information might encourage lifestyle changes that are difficult to sustain [91]. In addition, several previous studies showed higher stigma being associated with those diseases that were considered genetic [41, 42]. However, other studies suggest that strong negative long-term emotional responses to test results are rare and that people are generally poor at anticipating their emotional responses to future events (i.e., ‘affective forecasting’; [92]). In fact, the association in our study was only weak in individual countries and absent in the overall sample, suggesting that concern of finding out unwanted information is not a major factor in decisions about genetic testing.

Moreover, in the Russian sample the effect was weak and positive, suggesting that people with more concerns were actually more likely to undergo testing for research. Though small, this effect might be reflective of a general willingness to obtain some valid medical information regarding one’s health-related concerns irrespective of the source of such knowledge. This result again stresses the need for improvement of health-related literacy in the general population, including through genetic counselling. In the Era of Genome genetic counselling should be more readily available as it allows to support and educate people about medical, psychological and familial aspects of heritable diseases [93]. For example, there is evidence that genetic counselling can not only provide knowledge regarding a condition and potentially help to avoid a negative outcome (e.g. [57]) but also can improve perceived personal control and anxiety symptoms [94]. It is also worth noting that there was somewhat reduced variance for health-related concerns in the overall sample, as 90% of the sample answered “Yes” to one of the three questions that comprised this variable–“*I would rather not know of any potential debilitating diseases in my future*’.

The current study has a number of limitations. Firstly, the examined predictors explained only a small proportion of the overall variance in willingness to undergo genetic testing. This means that further research must explore additional factors and potential interactions among them. Secondly, some effects varied as a function of the sample. These differences across countries are likely to result from multiple factors that were beyond the current research, including differences in educational systems, socio-economic status, and media coverage of genetic-related questions (e.g. novel findings or regulations introduced). Some of these differences might stem from differences in recruitment strategies (e.g. targeting mostly student populations in Nigeria and Russia during data collection, which resulted in slightly larger samples for these two countries). Further, Nigerian and Russian sample were on average younger compared to UK and USA. However, the effect of age on willingness was very small and was not significant in a step-wise regression. Thirdly, the current study was correlational and did not allow causal inferences. Fourthly, only 4 out of the 86 countries were represented by substantial numbers of participants. As such, a truly cross-cultural study was beyond the scope of this paper. Another limitation might be posed by lengthy data collection, as it might introduce some uncontrolled confounds (e.g. media-related changes in attitudes or effects of Covid-19 pandemic). However, data in different countries were collected in parallel and most collection was made prior to the pandemic. Finally, the current study did not investigate willingness to undergo genetic testing beyond medical and research contexts, such as for obtaining information on non-medical traits, kinship and ancestry [95], and criminal investigations -–factors that will have increasing relevance as we progress further into the Genomic Era. Further research in this area would aid the development of new approaches to improve decision making about genetic testing [96].

## Conclusion

Genetic testing is at the forefront of discussions about healthcare, health insurance and disease prevention. Thus, it is important to investigate factors affecting decision-making regarding the willingness to undergo genetic testing. Our data showed positive attitudes towards genetic testing in the full sample and in separate countries. Genetic literacy, data protection and general trust in research institutions were among the robust predictors of willingness to undergo genetic testing. However, despite the large number of variables explored in the study, only a small proportion of variance in willingness to undergo genetic testing was explained. This suggests that decision making in this field is a product of many, potentially interacting, factors. Better understanding of the reasons for decisions regarding testing would help people to make decisions that are right for their specific circumstances.

## Supporting information

S1 Table Proportion of the total N and number of participants from a country after data cleaning. (DOCX)

S2 Table Post hoc comparisons for all study variables for Russia, Nigeria, USA and UK. (DOCX)
