# Supplementary material for: Attitudes towards genetic testing: The role of genetic literacy, motivated cognition, and socio-demographic characteristics
Source: PLoS One. 2023 Nov 15;18(11):e0293187. doi: 10.1371/journal.pone.0293187 (PMC10651000; doi:10.1371/journal.pone.0293187)
Supplement: S1 Table — (DOCX) [file pone.0293187.s001.docx]

| **Table S1 Proportion of the total N and number of participants from a country after data cleaning** | | | | | | | | | | | |
| --- | --- | --- | --- | --- | --- | --- | --- | --- | --- | --- | --- |
| **Country** | **Percentage** | **N** | **Country** | **Percentage** | **N** | **Country** | **Percentage** | **N** | **Country** | **Percentage** | **N** |
| **Russia** | 31.50 | 1358 | **Colombia** | 0.28 | 12 | **Portugal** | 0.09 | 4 | **Bahrain** | 0.02 | 1 |
| **Nigeria** | 23.66 | 1020 | **Finland** | 0.26 | 11 | **Albania** | 0.07 | 3 | **Bosnia and Herzegovina** | 0.02 | 1 |
| **USA** | 7.89 | 340 | **New Zealand** | 0.26 | 11 | **China** | 0.07 | 3 | **Costa Rica** | 0.02 | 1 |
| **UK** | 6.84 | 295 | **Poland** | 0.26 | 11 | **Egypt** | 0.07 | 3 | **Ecuador** | 0.02 | 1 |
| **Spain** | 5.94 | 256 | **Chile** | 0.23 | 10 | **Hong Kong (S.A.R.)** | 0.07 | 3 | **Ghana** | 0.02 | 1 |
| **Mexico** | 4.99 | 215 | **Greece** | 0.23 | 10 | **Hungary** | 0.07 | 3 | **Guatemala** | 0.02 | 1 |
| **Italy** | 3.92 | 169 | **Democratic People's Republic of Korea** | 0.19 | 8 | **Luxembourg** | 0.07 | 3 | **Iceland** | 0.02 | 1 |
| **Canada** | 1.58 | 68 | **India** | 0.19 | 8 | **Philippines** | 0.07 | 3 | **Indonesia** | 0.02 | 1 |
| **Argentina** | 1.44 | 62 | **Norway** | 0.19 | 8 | **Belarus** | 0.05 | 2 | **Iraq** | 0.02 | 1 |
| **Australia** | 1.11 | 48 | **Bulgaria** | 0.16 | 7 | **Cyprus** | 0.05 | 2 | **Namibia** | 0.02 | 1 |
| **Germany** | 0.90 | 39 | **Romania** | 0.16 | 7 | **Lebanon** | 0.05 | 2 | **Niger** | 0.02 | 1 |
| **Sweden** | 0.58 | 25 | **Switzerland** | 0.16 | 7 | **Malaysia** | 0.05 | 2 | **Palau** | 0.02 | 1 |
| **Ireland** | 0.51 | 22 | **Croatia** | 0.14 | 6 | **Nepal** | 0.05 | 2 | **Panama** | 0.02 | 1 |
| **Netherlands** | 0.42 | 18 | **Czech Republic** | 0.12 | 5 | **Oman** | 0.05 | 2 | **Qatar** | 0.02 | 1 |
| **Uruguay** | 0.39 | 17 | **Israel** | 0.12 | 5 | **Slovenia** | 0.05 | 2 | **Republic of Korea** | 0.02 | 1 |
| **France** | 0.37 | 16 | **Japan** | 0.12 | 5 | **Thailand** | 0.05 | 2 | **Saint Kitts and Nevis** | 0.02 | 1 |
| **Brazil** | 0.35 | 15 | **Serbia** | 0.12 | 5 | **Turkey** | 0.05 | 2 | **Slovakia** | 0.02 | 1 |
| **South Africa** | 0.32 | 14 | **Singapore** | 0.12 | 5 | **Afghanistan** | 0.02 | 1 | **Tunisia** | 0.02 | 1 |
| **Austria** | 0.28 | 12 | **Estonia** | 0.09 | 4 | **Algeria** | 0.02 | 1 | **Venezuela. Bolivarian Republic of...** | 0.02 | 1 |
| **Belgium** | 0.28 | 12 | **Peru** | 0.09 | 4 | **Antigua and Barbuda** | 0.02 | 1 | **Viet Nam** | 0.02 | 1 |
| Note: data on country of residence was not available for 75 participants (1.73 %) | | | | | | | | | | | |
