# Supplementary material for: Attitudes towards genetic testing: The role of genetic literacy, motivated cognition, and socio-demographic characteristics
Source: PLoS One. 2023 Nov 15;18(11):e0293187. doi: 10.1371/journal.pone.0293187 (PMC10651000; doi:10.1371/journal.pone.0293187)
Supplement: S2 Table — (DOCX) [file pone.0293187.s002.docx]

| **Table S2 Post Hoc Comparisons for all study variables for Russia, Nigeria, USA and UK** | | | | | | | | | | |
| --- | --- | --- | --- | --- | --- | --- | --- | --- | --- | --- |
| **Test for treatment** | | | | | | | | | | |
|  |  |  |  | Mean Difference | | SE |  | t |  | p**_bonf_** |
| USA |  | UK |  | -0.019 |  | 0.139 |  | -0.134 |  | 1.000 |
|  |  | Russia |  | 0.493 |  | 0.102 |  | 4.827 |  | < .001 |
|  |  | Nigeria |  | 0.663 |  | 0.106 |  | 6.243 |  | < .001 |
| UK |  | Russia |  | 0.511 |  | 0.114 |  | 4.469 |  | < .001 |
|  |  | Nigeria |  | 0.681 |  | 0.118 |  | 5.770 |  | < .001 |
| Russia |  | Nigeria |  | 0.170 |  | 0.071 |  | 2.382 |  | 0.104 |
| **Test for science** | | | | | | | | | | |
|  |  |  |  | Mean Difference | | SE |  | t |  | p**_bonf_** |
| USA |  | UK |  | -0.147 |  | 0.153 |  | -0.964 |  | 1.000 |
|  |  | Russia |  | 0.787 |  | 0.112 |  | 7.002 |  | < .001 |
|  |  | Nigeria |  | 0.637 |  | 0.117 |  | 5.446 |  | < .001 |
| UK |  | Russia |  | 0.934 |  | 0.126 |  | 7.417 |  | < .001 |
|  |  | Nigeria |  | 0.784 |  | 0.130 |  | 6.031 |  | < .001 |
| Russia |  | Nigeria |  | -0.150 |  | 0.079 |  | -1.910 |  | 0.337 |
| **Genetic Knowledge** | | | | | | | | | | |
|  |  |  |  | Mean Difference | | SE |  | t |  | p**_bonf_** |
| USA |  | UK |  | 0.051 |  | 0.012 |  | 4.367 |  | < .001 |
|  |  | Russia |  | 0.280 |  | 0.009 |  | 32.699 |  | < .001 |
|  |  | Nigeria |  | 0.236 |  | 0.009 |  | 26.453 |  | < .001 |
| UK |  | Russia |  | 0.229 |  | 0.010 |  | 23.886 |  | < .001 |
|  |  | Nigeria |  | 0.185 |  | 0.010 |  | 18.659 |  | < .001 |
| Russia |  | Nigeria |  | -0.044 |  | 0.006 |  | -7.404 |  | < .001 |
| **Data security** | | | | | | | | | | |
|  |  |  |  | Mean Difference | | SE |  | t |  | p**_bonf_** |
| USA |  | UK |  | 0.456 |  | 0.065 |  | 7.016 |  | < .001 |
|  |  | Russia |  | 0.713 |  | 0.048 |  | 14.912 |  | < .001 |
|  |  | Nigeria |  | 1.006 |  | 0.050 |  | 20.289 |  | < .001 |
| UK |  | Russia |  | 0.258 |  | 0.053 |  | 4.862 |  | < .001 |
|  |  | Nigeria |  | 0.550 |  | 0.055 |  | 10.082 |  | < .001 |
| Russia |  | Nigeria |  | 0.292 |  | 0.032 |  | 9.041 |  | < .001 |
| Health issues | | | | | | | | | | |
|  |  |  |  | Mean Difference | | SE |  | t |  | p**_bonf_** |
| USA |  | UK |  | 0.021 |  | 0.054 |  | 0.381 |  | 1.000 |
|  |  | Russia |  | 0.137 |  | 0.040 |  | 3.431 |  | 0.004 |
|  |  | Nigeria |  | 0.130 |  | 0.041 |  | 3.156 |  | 0.010 |
| UK |  | Russia |  | 0.116 |  | 0.044 |  | 2.630 |  | 0.052 |
|  |  | Nigeria |  | 0.110 |  | 0.045 |  | 2.414 |  | 0.095 |
| Russia |  | Nigeria |  | -0.006 |  | 0.027 |  | -0.236 |  | 1.000 |
| **Religiosity** | | | | | | | | | | |
|  |  |  |  | Mean Difference | | SE |  | t |  | p**_bonf_** |
| USA |  | UK |  | 0.756 |  | 0.242 |  | 3.124 |  | 0.011 |
|  |  | Russia |  | 0.048 |  | 0.174 |  | 0.278 |  | 1.000 |
|  |  | Nigeria |  | -3.649 |  | 0.180 |  | -20.264 |  | < .001 |
| UK |  | Russia |  | -0.708 |  | 0.202 |  | -3.498 |  | 0.003 |
|  |  | Nigeria |  | -4.406 |  | 0.208 |  | -21.206 |  | < .001 |
| Russia |  | Nigeria |  | -3.698 |  | 0.122 |  | -30.433 |  | < .001 |
| **Destiny is written** | | | | | | | | | | |
|  |  |  |  | Mean Difference | | SE |  | t |  | p**_bonf_** |
| USA |  | UK |  | 0.102 |  | 0.137 |  | 0.742 |  | 1.000 |
|  |  | Russia |  | 0.213 |  | 0.101 |  | 2.105 |  | 0.212 |
|  |  | Nigeria |  | 0.724 |  | 0.105 |  | 6.892 |  | < .001 |
| UK |  | Russia |  | 0.111 |  | 0.113 |  | 0.978 |  | 1.000 |
|  |  | Nigeria |  | 0.622 |  | 0.117 |  | 5.325 |  | < .001 |
| Russia |  | Nigeria |  | 0.511 |  | 0.071 |  | 7.242 |  | < .001 |
| **Mistrust to research** | | | | | | | | | | |
|  |  |  |  | Mean Difference | | SE |  | t |  | p**_bonf_** |
| USA |  | UK |  | 0.844 |  | 0.143 |  | 5.909 |  | < .001 |
|  |  | Russia |  | 0.122 |  | 0.105 |  | 1.164 |  | 1.000 |
|  |  | Nigeria |  | -0.339 |  | 0.109 |  | -3.101 |  | 0.012 |
| UK |  | Russia |  | -0.722 |  | 0.118 |  | -6.130 |  | < .001 |
|  |  | Nigeria |  | -1.183 |  | 0.122 |  | -9.735 |  | < .001 |
| Russia |  | Nigeria |  | -0.461 |  | 0.073 |  | -6.276 |  | < .001 |
| **Age** | | | | | | | | | | |
|  |  |  |  | Mean Difference | | SE |  | t |  | p**_bonf_** |
| USA |  | UK |  | -5.084 |  | 0.659 |  | -7.719 |  | < .001 |
|  |  | Russia |  | 18.646 |  | 0.485 |  | 38.439 |  | < .001 |
|  |  | Nigeria |  | 16.169 |  | 0.507 |  | 31.903 |  | < .001 |
| UK |  | Russia |  | 23.729 |  | 0.542 |  | 43.756 |  | < .001 |
|  |  | Nigeria |  | 21.253 |  | 0.562 |  | 37.827 |  | < .001 |
| Russia |  | Nigeria |  | -2.477 |  | 0.342 |  | -7.234 |  | < .001 |
| **Sex** | | | | | | | | | | |
|  |  |  |  | Mean Difference | | SE |  | t |  | p**_bonf_** |
| USA |  | UK |  | -0.058 |  | 0.038 |  | -1.509 |  | 0.789 |
|  |  | Russia |  | -0.248 |  | 0.028 |  | -8.822 |  | < .001 |
|  |  | Nigeria |  | -0.250 |  | 0.029 |  | -8.576 |  | < .001 |
| UK |  | Russia |  | -0.190 |  | 0.031 |  | -6.041 |  | < .001 |
|  |  | Nigeria |  | -0.193 |  | 0.032 |  | -5.937 |  | < .001 |
| Russia |  | Nigeria |  | -0.003 |  | 0.020 |  | -0.137 |  | 1.000 |
| **Education level** | | | | | | | | | | |
|  |  |  |  | Mean Difference | | SE |  | t |  | p**_bonf_** |
| USA |  | UK |  | -0.050 |  | 0.066 |  | -0.754 |  | 1.000 |
|  |  | Russia |  | 0.519 |  | 0.049 |  | 10.646 |  | < .001 |
|  |  | Nigeria |  | 0.027 |  | 0.051 |  | 0.524 |  | 1.000 |
| UK |  | Russia |  | 0.568 |  | 0.054 |  | 10.469 |  | < .001 |
|  |  | Nigeria |  | 0.076 |  | 0.056 |  | 1.363 |  | 1.000 |
| Russia |  | Nigeria |  | -0.492 |  | 0.034 |  | -14.511 |  | < .001 |
| Note:  SE – standard error; Mean difference = score of the reference country (first column) minus score of the comparison country (second column). | | | | | | | | | | |
